# CPC-ETC1 chimeric protein localization data in *Arabidopsis* root epidermis

**DOI:** 10.1016/j.dib.2018.04.055

**Published:** 2018-04-25

**Authors:** R. Tominaga-Wada, T. Wada

**Affiliations:** Graduate School of Biosphere Sciences, Hiroshima University, 1-4-4 Kagamiyama, Higashi-Hiroshima 739-8528, Japan

## Abstract

Intercellular movement of transcription factor proteins is essential for plant development. The R3 type MYB transcription factor protein, CAPRICE (CPC), moves from non-hair cells to root-hair cells where it promotes root hair formation in *Arabidopsis* root epidermis. In contrast, the CPC homolog of ENHANCER OF TRY AND CPC1 (ETC1) cannot move in root epidermal cells. In this work, we present protein localization data of CPC-ETC1 chimeric proteins. Localization of CPC-ETC1-GFP fusion proteins of chimera1 and chimera2 transgenic plants was observed using confocal laser scanning microscope. Insertion of ETC1-specific amino acids into CPC somewhat prevents normal protein localization of CPC in root epidermal cells. Cell-to-cell movement of chimera1 and chimera2 proteins from non-hair cells to root-hair cells was interfered. Nuclear localization was also inhibited, especially in chimera1.

**Specifications table**

**Table:** 

Subject area	Biology
More specific subject area	Plant Sciences
Type of data	Figure
How data was acquired	Confocal laser scanning microscope (Zeiss LSM-510 Meta)
Data format	Raw
Experimental factors	–
Experimental features	–
Data source location	Higashi-Hiroshima, Japan
Data accessibility	Data are presented in this article
Related research article	Effect of amino acid substitution of CAPRICE on cell-to-cell movement ability in *Arabidopsis* root epidermis, *Developmental Biology*, in press.

**Value of the data**

• The data provide information about the protein localization and cell-to-cell movement properties of CPC-ETC1 chimeric proteins in *Arabidopsis* root epidermal cells.
• This study shows the importance of precise amino acid sequence of CPC in proper cell-to-cell movement ability in *Arabidopsis* root epidermal cells.
• The cell-to-cell movement data of chimera proteins in *Arabidopsis* root epidermis helps to understand the functions of R3-type MYB transcription factors.

## 1 Data

**Fig. 1:**
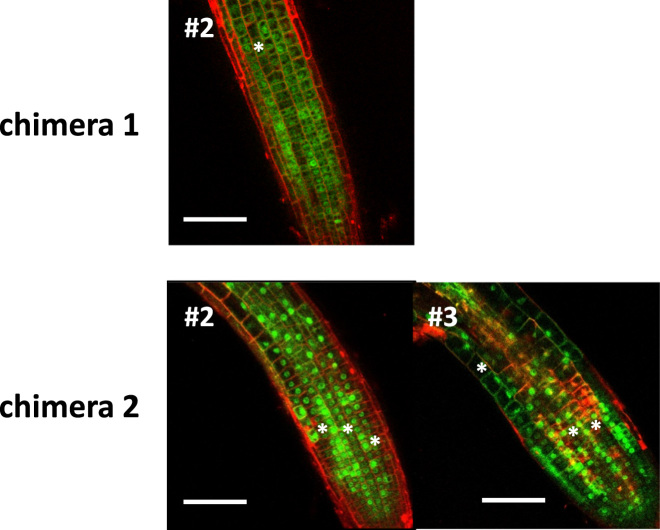
Distribution of GFP fluorescence in the transgenic *Arabidopsis* plants expressing Chimera 1 and Chimera 2 constructs. Homozygous transgenic lines of Chimera 1#2, Chimera 2#2, and Chimera 2#3 are shown. Confocal laser scanning microscope images showing GFP (green) and propidium iodide (red) fluorescence in the root epidermis of five-day-old seedlings. Asterisks indicate the root hair cell files. Scale bars: 100 µm.

Fig. 1 shows the localization of CPC-ETC1 chimera-GFP fusion proteins in *Arabidopsis* root epidermis. The level of GFP fluorescence was slightly lower in root hair cells than in non-hair cells of all transgenic plants of Chimera 1#2, Chimera 2#2, and Chimera 2#3 in this study. Clear nucleus localization of the GFP fusion protein was not observed in Chimera 1#2 transgenic epidermal cells.

## 2 Experimental design, materials and methods

### 2.1 Plant material and growth conditions

This study utilized previously reported transgenic *Arabidopsis thaliana* (L.) Heynh. lines CPC-ETC1 Chimera 1 #2, Chimera 2 #2, and Chimera 2 #3 [1] of the ecotype Columbia (Col-0). Seeds were surface-sterilized and sown on 1.5% agar plates as described previously [2]. The plates with sawn seeds were kept at 4 °C for 2 days and then incubated at 22 °C under constant white light (50–100 µmol m^−2^ s^−1^). For each transgenic line, five-day-old seedlings were examined for the GFP fused chimeric protein localization.

### 2.2 Gene constructs

Gene constructs for CPC-ETC1 chimeric proteins were generated in the *CPCp:CPC:2xGFP* backbone [3] by TaKaRa (TaKaRa, Japan). To create the Chimera 1 construct, *ETC1*-specific DNA sequence corresponding to the NT amino acid sequence was inserted into the *CPC* coding region between the 11th (D) and 12th (K) position of the CPC amino acid sequence in *CPCp:CPC:2xGFP* [1]. To create the Chimera 2 construct, *ETC1*-specific DNA sequence corresponding to the HLKTNPTIV amino acid sequence was inserted into the *CPC* coding region between the 21st (K) and 22nd (A) position of the CPC amino acid sequence in *CPCp:CPC:2xGFP* [1].

### 2.3 Transgenic plants

The floral dip method was used for the plant transformation in this study [4], and the transgenic plants were selected on 0.5× Murashige and Skoog's agar plates containing 50 mg/L kanamycin. The homozygous transgenic lines were selected for kanamycin resistance.

### 2.4 Microscopy

For each transgenic line of Chimera 1#2, Chimera 2#2, and Chimera 2#3, five-day-old seedling roots were analyzed for GFP fluorescence. The transgenic GFP fusion lines were stained with 5 µg/mL propidium iodide for 30 s and then washed with water. Confocal images were obtained with a Zeiss LSM-510 Meta confocal laser scanning microscope using 488-nm laser lines for GFP excitation. Image processing was performed with Photoshop version 7.0 (Adobe Systems, CA, USA).
