# Supplementary figures and images for: CPC-ETC1 chimeric protein localization data in Arabidopsis root epidermis
Source: Data Brief. 2018 Apr 25;18:1773–6. doi: 10.1016/j.dib.2018.04.055 (PMC5997977; doi:10.1016/j.dib.2018.04.055)

**chimera 1**

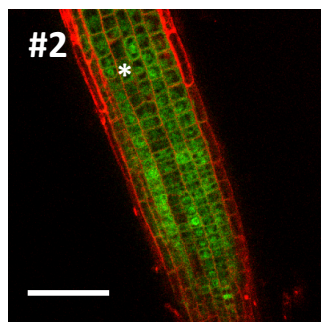

**chimera 2**

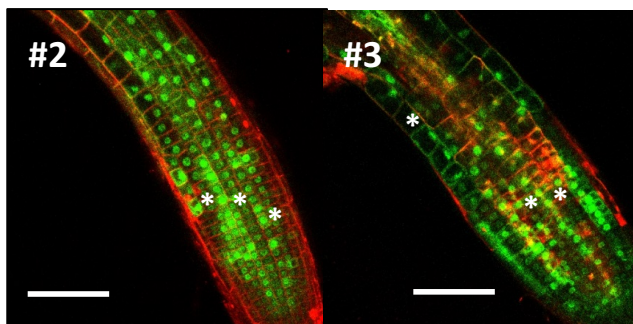

Supplement: Supplementary file 2 — Supplementary material [file mmc2.pdf]
